# Comparable Accuracies of Nonstructural Protein 1- and Envelope Protein-Based Enzyme-Linked Immunosorbent Assays in Detecting Anti-Dengue Immunoglobulin G Antibodies

**DOI:** 10.3390/diagnostics11050741

**Published:** 2021-04-21

**Authors:** Jedhan Ucat Galula, Gielenny M. Salem, Raul V. Destura, Roland Remenyi, Day-Yu Chao

**Affiliations:** 1 Graduate Institute of Microbiology and Public Health, College of Veterinary Medicine, National Chung Hsing University, Taichung 402, Taiwan; creepingeruption@hotmail.com (J.U.G.); gielennymsalem@yahoo.com.ph (G.M.S.); 2 Institute of Molecular Biology and Biotechnology, National Institutes of Health, University of the Philippines Manila, Manila 1000, Philippines; rvdestura@up.edu.ph; 3 Biomedical Research Unit, Clinical and Translational Research Institute, The Medical City, Pasig 1605, Philippines; rgremenyi@themedicalcity.com

**Keywords:** dengue virus, NS1 protein, E protein, GAC–ELISA, indirect IgG ELISA, composite reference standard

## Abstract

Background: Dengue virus (DENV) infection remains a global public health concern. Enzyme-linked immunosorbent assays (ELISAs), which detect antibodies targeting the envelope (E) protein of DENV, serve as the front-line serological test for presumptive dengue diagnosis. Very few studies have determined the serostatus by detecting antibodies targeting the nonstructural protein 1 (NS1), which can function as diagnostic biomarkers to distinguish natural immunity from vaccine-induced immunity. Methods: We used community-acquired human serum specimens, with the serostatus confirmed by focus reduction microneutralization test (FRμNT), to evaluate the diagnostic performances of two NS1-based ELISA methods, namely, immunoglobulin G antibody-capture ELISA (NS1 GAC–ELISA) and indirect NS1 IgG ELISA, and compared the results with an E-based virus-like particle (VLP) GAC–ELISA. Results: NS1-based methods had comparable accuracies as VLP GAC–ELISA. Although the sensitivity in detecting anti-NS1 IgM was poor, indirect NS1 IgG ELISA showed similar limits of detection (~1–2 ng/mL) as NS1 GAC–ELISA in detecting anti-NS1 IgG. Combining the results from two or more tests as a composite reference standard can determine the DENV serostatus with a specificity reaching 100%. Conclusion: NS1-based ELISAs have comparable accuracies as VLP GAC–ELISA in determining dengue serostatus, which could effectively assist clinicians during assessments of vaccine eligibility.

## 1. Introduction

Flaviviruses of the family *Flaviviridae* are associated with several arthropod-borne diseases that are divided into different serological complexes, including members of the tick-borne encephalitis virus (TBEV), yellow fever virus (YFV), Japanese encephalitis virus (JEV), Zika virus (ZIKV), and dengue virus (DENV) serocomplexes based on the antibodies against the immunodominant envelope (E) protein [1,2]. DENV, made up of four antigenically distinct serotypes (DENV1 to 4), remains the mosquito-borne flavivirus that continually imposes the highest economic and public health burden, particularly in tropical and subtropical countries [3,4,5]. Infection with any of the serotypes can cause a broad spectrum of clinical manifestations, ranging from asymptomatic or mild febrile symptoms dengue fever to rarely life-threatening dengue hemorrhagic fever or dengue shock syndrome. It is estimated that 390 million DENV infections occur annually worldwide, with 500,000 severe cases and 25,000 deaths, mainly affecting children [6]. To date, commercial vaccines for human use are only available for TBEV (FSME-IMMUN^®^, Encepur^®^, TBE-Moscow^®^, and EnceVir^®^), YFV (17D and YF-Vax^®^), JEV (IXIARO^®^ and IMOJEV^®^), and DENV (Dengvaxia^®^), despite the steady expansion and circulation of flaviviruses worldwide [7,8,9]. Corollary to this, it is imperative to establish a reliable serological assay that can differentiate natural immunity from vaccine-induced immunity. This serological differentiation is essential because the demonstration of natural infection in vaccinated populations is crucial for monitoring and evaluating vaccine efficacy and safety.

Flaviviruses are enveloped viruses with a single-stranded, positive-sense RNA genome of approximately 11 kb length [1,10]. Their genome encodes a single polyprotein that is co- and posttranslationally processed into three structural (capsid [C], premembrane [prM], and envelope [E]), and seven nonstructural (NS1, NS2a, NS2b, NS3, NS4a, NS4b, and NS5) proteins [1,10]. The highly conserved NS1 glycoprotein is about 352 amino acids long with a molecular weight of 46 to 55 kDa [11]. NS1 proteins exist in different forms [12], namely, (1) dimers that retain inside the cytoplasm; (2) membrane-bound, interacting with the host proteins and viral RNA replication machinery; or (3) soluble hexamers secreted into the extracellular space. The soluble NS1 proteins released from infected cells are highly immunogenic and can elicit substantial levels of specific antibodies [13,14,15]. Hence, previous studies have proposed to use antibodies to NS1 as surrogate serological biomarkers in distinguishing immunity due to natural infection from vaccination in populations administered with TBEV [16], JEV [17,18,19], and DENV [20,21,22,23] vaccines using an indirect enzyme-linked immunosorbent assay (indirect anti-NS1 ELISA) method.

The conventional three-layer format indirect anti-NS1 ELISAs require either the sensitization of a microplate with purified NS1 antigens [21,22,24] or the capture of an unpurified NS1 antigen by NS1-specific murine monoclonal antibodies (MAbs) [19,25] or rabbit polyclonal sera [26] before the addition of test serum specimens. Another approach used to detect NS1-specific antibodies is the five-layer format immunoglobulin M or G (IgM/IgG)-capture ELISA (MAC/GAC–ELISA) [27,28,29,30]. Comparatively, the indirect anti-NS1 ELISA is more cost effective and has a faster turnaround time, whereas the MAC/GAC–ELISA does not need a purified antigen but is more time consuming and requires an additional detector antibody. Additionally, our previous studies suggest that the sensitivity of NS1 MAC/GAC–ELISAs could be significantly enhanced upon predepletion of anti-prM/E antibodies in flavivirus-infected patients’ sera [28,29,30]. Our unpublished data also showed that indirect anti-NS1 ELISA has lower sensitivity in detecting anti-NS1 IgM than using MAC–ELISA due to the relative abundance of anti-prM/E antibodies during acute DENV infection. However, whether this observation can be applied to detect anti-NS1 IgG in the community serum samples after recovery from DENV infection for years require further investigation. Thus far, such a direct comparison of the sensitivity between anti-NS1 immunoglobulin capture and indirect anti-NS1 ELISAs is still missing. Using archived DENV-infected clinical and community serum panels, this study aimed to establish (1) the sensitivity and specificity of the anti-dengue NS1 GAC–ELISA, compared to the indirect NS1 IgG ELISA; (2) the lower threshold of antibody concentration for detection; and (3) a composite reference standard that can be used to reliably determine the true serostatus of dengue infection in the absence of a neutralization test (NT) as the gold standard. In the future, such a composite reference standard could aid clinicians in their decision-making process concerning a patient’s eligibility for Dengvaxia^®^ vaccination.

## 2. Materials and Methods

### 2.1. Cells and Antibodies

COS-1 (CRL-1650; ATCC, Manassas, VA, USA) and HEK293T (CRL-3216; ATTC, Manassas, VA, USA) cells were grown at 37 °C with 5% CO_2_ in Dulbecco’s modified Eagle’s minimal essential medium (DMEM; Gibco, Grand Island, NY, USA) supplemented with 10% heat-inactivated fetal bovine serum (FBS; HyClone Laboratories, Inc., Logan, UT, USA), 110 mg/L sodium pyruvate, 0.1 mM nonessential amino acids, 2 mM l-glutamine, 20 mL/L 7.5% NaHCO3, 100 U/mL penicillin, and 100 μg/mL streptomycin.

Serotype-specific anti-DENV VLP rabbit sera and murine hyperimmune ascitic fluid (MHIAF) were kindly provided by Dr. G.-J. Chang (Diagnostic and Reference Laboratory, DVBD-CDC, Fort Collins, CO, USA). Rabbit anti-His polyclonal serum was purchased from Yao-Hong Biotechnology, Inc., Taipei, Taiwan.

### 2.2. Plasmid Construction, Protein Expression, and Purification

The previously described transcriptionally and translationally optimized eukaryotic cell expression plasmids [31,32] were used as the vector to transiently express dengue virus soluble NS1 proteins or premembrane/envelope (prM/E) proteins, which self-assemble to form virus-like particles (VLPs), following standard molecular cloning procedures. The NS1 protein expression plasmids were modified to contain a 6×-Histidine tag at the carboxyterminal segment using the primers listed in Supplementary Table S1 with QuikChange II site-directed mutagenesis kit (Stratagene, La Jolla, CA, USA).

For transient expressions of NS1 proteins and VLPs, electroporation of COS-1 cells was carried out as previously described [33] with the recombinant expression plasmids encoding the NS1 or prM/E genes from DENV1 strain 16007, DENV2 strain 16681, DENV3 strain C0331/94, and DENV4 strain H241 (kindly provided by Dr. G.-J. Chang, Diagnostic and Reference Laboratory, DVBD-CDC, USA). Electroporated cells were recovered in 50 mL culture medium, seeded in separate 150 cm^2^ culture flasks for NS1 and VLP expressions, and incubated at 28 °C with 5% CO_2_. This low-temperature cultivation helps maintain longer cell viability while inhibiting cell growth and has been positively correlated with increased recombinant protein production in several mammalian cell lines [34]. Five days later, the recombinant antigens were harvested, clarified, and filtered using 0.20 μM syringe filters (Minisart; Sartorius, Gottingen, Germany).

The NS1-containing tissue culture supernatants were purified by affinity chromatography. Briefly, samples were diluted with 10 mM imidazole in binding buffer (0.1 M Tris-base, 0.5 M NaCl, pH 8.0), loaded onto a Ni^2+^ affinity column (Chelating Sepharose Fast Flow; GE Healthcare, Uppsala, Sweden), and washed with 50 mM imidazole in the binding buffer. Finally, bound NS1 proteins were eluted with 500 mM imidazole in the binding buffer. Fractions containing the purified NS1 proteins were pooled and dialyzed with phosphate-buffered saline (PBS, pH 7.4) using a 10K MWCO centrifugal filter device (Amicon Ultra 15; Merck, Darmstadt, Germany), and stored at −80 °C in aliquots until further use. The purified protein concentrations were determined using the Bradford reagent (Bio-Rad, Hercules, CA, USA) and bovine serum albumin (New England BioLabs, Inc., Ipswich, MA, USA) as the protein standard.

Commercial NS1 proteins from DENV1 strain Nauru/Western Pacific/1974, DENV2 strain Thailand/16681/84, DENV3 strain Sri Lanka D3/H/IMTSSA/SRI/2000/1266, and DENV4 strain Dominica/814669/1981 were purchased from the Native Antigen Company (Oxford, Oxfordshire, UK), now acquired by LGC Group.

### 2.3. Sodium Dodecyl Sulfate–Polyacrylamide Gel Electrophoresis (SDS–PAGE) and Western Blotting

Purified proteins (1 µg) were mixed with a nonreducing Laemmli sample buffer, resolved on a 4–20% gradient polyacrylamide gel (Mini-Protean TGX; Bio-Rad, Hercules, CA, USA) in Tris-glycine SDS running buffer, and stained with Coomassie blue dye. For immunodetection, proteins were electrophoretically transferred onto a polyvinylidene difluoride (PVDF) membrane followed by incubation with blocking buffer (5% skimmed milk in PBS with 0.1% Tween 20 [0.1% PBS-T]) for 1 h at room temperature (RT). The membrane was hybridized with MAb mhFL0221 at 1 µg/mL. Details of MAb mhFL0221 are described in Section 2.4. Bound MAbs were detected by adding horseradish peroxidase (HRP)-conjugated donkey anti-human IgG (Jackson ImmunoResearch, Westgrove, PA, USA). After washing with 0.1% PBS-T, signals were developed using an enhanced chemiluminescence substrate (Super Signal West Pico PLUS; Thermo Fisher Scientific, Rockford, IL, USA). The image was captured using ImageQuant LAS 4000 (GE Healthcare, Uppsala, Sweden).

### 2.4. Generation of Stable Cell Lines

Cell lines constitutively expressing each of the four serotypes of DENV soluble NS1 proteins were generated via CRISPR/Cas9-mediated gene knock-in system using the Genome-CRISPR Human AAVS1 Safe Harbor Gene Knock-In kit (GeneCopoeia Inc., Rockville, MD, USA). Briefly, the NS1 gene inserts of the pCBD-NS1-His expression plasmids were PCR-amplified using the primers listed in Table 1 and subcloned into the AAVS1 donor plasmid (SH200) using the NEBuilder HiFi DNA Assembly Master Mix kit (New England BioLabs, Inc., Ipswich, MA, USA). HEK293T cells seeded at 2.5 × 10^5^ cells/mL onto 12-well cell culture plates (Costar; Corning Inc., Corning, NY, USA) were cotransfected with 800 ng each of the AAVS1 CRISPR/Cas9 plasmid (SH100) and SH200-NS1 donor plasmid using Lipofectamine 2000 (Invitrogen; Thermo Fisher Scientific, Carlsbad, CA, USA). Transfected cells were maintained in nonselective culture media for 48 h and then treated with 10 μg/mL of Puromycin (Cyrusbioscience Inc., New Taipei, Taiwan) for two weeks. Monoclonal cells were isolated from the puromycin-resistant colonies by limiting dilution in puromycin-containing media. We determined the stable expression of the NS1 proteins by antigen-capture ELISA (Ag–ELISA) after 10 consecutive passages.

A cell line stably expressing MAb mhFL0221 was also in-house generated from HEK293T cells following the same CRISPR/Cas9-mediated gene knock-in system and cultured in serum-free media (HyClone SFM4MegaVir; Cytiva, Logan, UT, USA). MAb mhFL0221, initially derived from a pan-flavivirus anti-NS1 murine MAb FL0221 (a kind gift from Dr. L.-K. Chen, Tzu Chi University Hospital, Hualien, Taiwan), was engineered to be a chimeric antibody by replacing the original murine IgG2a constant heavy and kappa light chain regions with those of the human IgG1.

### 2.5. Antigen Titration by Ag–ELISA

To standardize the VLP antigen concentration, an Ag–ELISA was performed with twofold titration of the VLP-containing tissue culture supernatants as previously described [35]. Briefly, 96-well plates were coated overnight at 4 °C with serotype-specific anti-DENV VLP rabbit sera at 1:500 dilution in coating buffer (0.015 M Na_2_CO_3_, 0.035 M NaHCO_3_; pH 9.6), followed by incubation with blocking buffer for 1 h at 37 °C. The VLP antigens were titrated twofold and added to wells in duplicate, incubated for 1 h at 37 °C, and washed with 0.1% PBS-T. Captured VLPs were detected with serotype-specific MHIAF at 1:2000 dilution in blocking buffer, incubated for 1 h at 37 °C, and washed. The MHIAFs were detected with an HRP-conjugated goat anti-mouse IgG (Jackson ImmunoResearch, Westgrove, PA, USA) at 1:5000 dilution in blocking buffer, incubated for 1 h at 37 °C, and washed. The bound conjugate was detected with 3,3′,5,5′-tetramethylbenzidine (TMB) substrate (Neogen Corp., Lexington, KY, USA) for 10 min at RT, stopped the reaction with 2N H_2_SO_4_, and measured the optical density at 450 nm (OD450) with a reference wavelength of 630 nm using a Tecan Sunrise microplate reader (Tecan, Grödig, Austria). The VLP concentration was standardized to an OD450 of 1.0, within the region of antigen excess near the sigmoidal dilution curve’s upper asymptote as modeled using a four-parameter logistic (4-PL) log fit equation, which is ~25 ng, as we have previously determined [30,35,36]. The purified NS1 proteins were similarly titrated at varying concentrations to determine the optimal antigen amount for detecting anti-NS1 antibodies using rabbit anti-His polyclonal serum and serotype-specific MHIAFs as capture and detector antibodies, respectively.

### 2.6. NS1- and VLP-Specific MAC/GAC–ELISAs

The presence of NS1- and E-specific IgM or IgG antibodies in human serum samples were determined through NS1 and VLP MAC/GAC–ELISAs, respectively, as previously described [28,29,30]. Briefly, 96-well plates were coated overnight at 4 °C with goat anti-human IgM or IgG (Kirkegaard & Perry Laboratories, Gaithersburg, MD, USA) at 1:2000 dilution in coating buffer followed by incubation with blocking buffer for 1 h at 37 °C. A volume of 50 microliters of infected patient sera, including negative control serum at 1:2000 dilution in blocking buffer, were added to wells in duplicate and incubated for 1 h at 37 °C. After diluting standardized VLP antigens in blocking buffer, we tested against each serum sample and detected with the homologous MHIAFs.

To predeplete the anti-prM/E antibodies in the serum specimens for NS1 MAC/GAC–ELISA, the antigen-capture method was used to eliminate VLP immunocomplexes in 96-well ELISA plates as previously described [28,29,30]. Briefly, 50 μL of the patient sera and negative control serum diluted 1:2000 in PBS premixed with a combination of DENV2 + 3 or DENV1–4 VLP antigens were added to wells coated with the homologous rabbit anti-DENV VLP sera, incubated for 1 h at 37 °C, and then transferred to the plates precoated with anti-human IgG for NS1 MAC/GAC–ELISA, as described above. Notably, we previously demonstrated that this predepletion step could enhance the assay sensitivity for up to ~10 fold [28,29].

### 2.7. Indirect NS1 IgM/IgG ELISA

To detect the presence of NS1-specific IgG antibodies in human serum samples through an indirect NS1 IgM or IgG ELISA, we coated 96-well plates overnight at 4 °C with pooled DENV1 to 4 NS1 antigens (at an equimolar amount of 16 ng) in coating buffer and incubated with for 1 h at 37 °C. A total volume of 50 microliters of infected patient sera, including negative control serum at 1:500 dilution in blocking buffer, were added to wells in duplicate, incubated for 1 h at 37 °C, and washed with 0.1% PBS-T. Anti-NS1 IgM and IgG antibodies were detected with HRP-conjugated goat anti-human IgM and IgG (Jackson ImmunoResearch, Westgrove, PA, USA), respectively, at 1:5000 dilution in blocking buffer, incubated for 1 h at 37 °C, and washed. We detected bound conjugate by adding TMB substrate for 10 min at RT, stopped the reaction with 2N H_2_SO_4_, and measured the absorbance at OD450 with a reference wavelength of 630 nm. Before performing the indirect NS1 IgM ELISA, human serum specimens were preincubated in 96-well ELISA plates coated with goat anti-human IgG at 1:500 dilution to deplete potentially interfering IgG antibodies.

### 2.8. Limits of Detection of NS1 GAC- and Indirect NS1 IgG ELISAs

To determine the limits of detection of NS1 GAC- and indirect NS1 IgG ELISAs, we purified MAb mhFL0221 and negative control serum using Protein G columns (HiTrap Protein G HP; GE Healthcare, Uppsala, Sweden) following the manufacturer’s protocol. The IgG concentrations were determined using the Bradford reagent (Bio-Rad, Hercules, CA, USA) and known concentrations of a purified total human IgG (in-house produced) as IgG standard.

We performed the NS1 GAC- and indirect NS1 IgG ELISAs as described above, using a combination of DENV2 + 3 or DENV1–4 NS1 antigens, with 12-point serial twofold dilutions (from 1000–0.5 ng/mL) of MAb mhFL0221 and negative control IgGs as detector antibodies. The limit of detection for each assay, defined as the minimum detectable concentration (MDC), was estimated from a 4-PL log fit model applied to the standard curve data [37]. The MDC is the IgG concentration corresponding to the lower asymptote’s interpolated intersection of the upper 95% confidence interval (95% CI) with the 4-PL log fit of the standard curve data. 

### 2.9. Human Serum Panel

Human serum specimens, with the status of infection confirmed by a 90% focus reduction microneutralization test (FRμNT90) or reverse transcription–polymerase chain reaction (RT–PCR), were obtained from the dengue clinical cohort study of Kaohsiung Medical University, Taiwan, and the community volunteers of National Chung Hsing University, Taiwan. The use of these serum specimens was reviewed and approved by the Institutional Review Board (IRB) of Kaohsiung Medical University (IRB No. KMUHIRB-E (II)-20180092). Each patient or his/her legal guardian/s provided written informed consent, whereas all community volunteers provided assent following an explanation of the study’s purpose. All data were anonymized.

As shown in Table 1, 61 FRμNT90-confirmed late (>3 months to years postsymptom onset [PSO]) convalescent-phase dengue serum specimens were assembled as the target disease panel for NS1 and VLP GAC–ELISAs, and indirect NS1 IgG ELISA, including 20 primary dengue infections and 41 repeated dengue infections, herein termed secondary infections. Additional 31 RT–PCR-confirmed primary acute-phase (<7 days PSO) to early (<3 months PSO) and late convalescent-phase serum specimens were also assembled for NS1 and VLP MAC–ELISAs, and NS1 IgM indirect ELISA. Overall, 51 normal human serum specimens were included as dengue-negative controls.

### 2.10. Data Processing and Statistical Analysis

The positive-to-negative (P/N) ratios, based on the mean OD450 values of duplicate samples from each patient serum (P) and negative control serum (N) reacted with the VLP or NS1 antigens, were calculated for the interpretation of test results. Both positive and negative serum controls were included in each ELISA plate for test validation.

Data were expressed as means ± standard deviations. One-way analysis of variance (ANOVA) was used for the comparison of data sets across multiple groups. A fitted receiver operating characteristic (ROC) curve analysis was used to compare diagnostic assay performances. To quantify the accuracy of discrimination, we used the area-under-the-ROC curve (AUC). We also calculated the positive likelihood ratio (LR^+^) with the following formula: LR^+^ = sensitivity/(100 – specificity); this calculation allowed us to determine the optimal P/N ratio cutoff. With a significance level set at *p* < 0.05, all statistical analyses were performed using GraphPad Prism version 6.0 (GraphPad Software, San Diego, CA, USA).

## 3. Results

### 3.1. Confirmation of In-House NS1 Protein Quality

**Figure 1:**
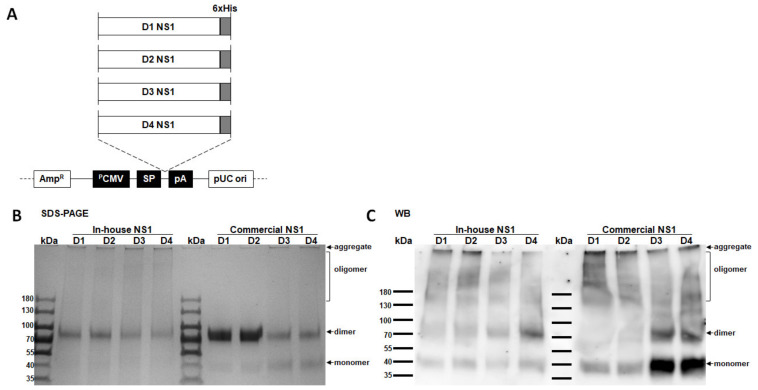
Map of plasmid constructs expressing nonstructural protein 1 (NS1) proteins from four dengue virus (DENV) serotypes and comparison between in-house- and commercially produced NS1 proteins. (**A**) Schematic representation of the plasmid constructs encoding different NS1 protein genes from DENV serotypes 1 (strain 16007), 2 (strain 16681), 3 (strain C0331/94), and 4 (strain H241) with 6x-Histidine tag at the carboxyterminal gene segment. The plasmid vector backbone contained the Ampicillin resistance gene (AmpR), human cytomegalovirus early gene promoter (PCMV), Japanese encephalitis virus signal peptide (SP), bovine growth hormone poly(A) signal (pA), and pUC origin of replication (pUC ori). (**B**,**C**) NS1 proteins from DENV1 to 4 (D1 to D4) were produced in-house or commercially obtained from the Native Antigen Company. (**B**) Visualization of nonreducing sodium dodecyl sulfate–polyacrylamide gel electrophoresis (SDS–PAGE) by Coomassie blue staining confirmed the presence of NS1 proteins with comparable molecular weights. (**C**) Western blot using the pan-flavivirus anti-NS1 MAb mhFL0221 demonstrating the oligomeric, dimeric, and monomeric forms of the soluble NS1 proteins in both in-house- and commercially produced antigens under nonreducing SDS–PAGE.

To compare the sensitivities of NS1 GAC- and indirect NS1 IgG ELISAs properly, (1) the use of high-quality purified recombinant NS1 proteins and (2) normalization of the antigens are essential. Therefore, we first set out to reclone the previously generated expression plasmid vectors expressing NS1 proteins from all four serotypes of DENV; recloning consisted of adding a 6×-His tag at the C-terminal of NS1 genes for downstream protein purification (Figure 1A). Upon reviewing the relevant literature, we found that the commercially available NS1 proteins from Native Antigen Company are widely used in several NS1 protein-related studies and were also used as the standard antigens in this study to ensure the quality of our in-house-produced NS1 proteins. Upon visualization by Coomassie blue staining of the nonreducing SDS–PAGE (Figure 1B), the in-house and commercial NS1 proteins appeared to exist as heterogeneous conformations (as evident by the broad smears) ranging from monomers, dimers, oligomers, and as protein aggregates that were not able to migrate into the resolving gel due to their large size. Western blotting result (Figure 1C) of the recombinant proteins from both sources further showed a migration pattern consistent with the molecular mass of the oligomeric, dimeric, monomer, and aggregated forms. In summary, our in-house-generated NS1 proteins from all four DENV serotypes showed comparable purity with the commercial antigens.

**Figure 2:**
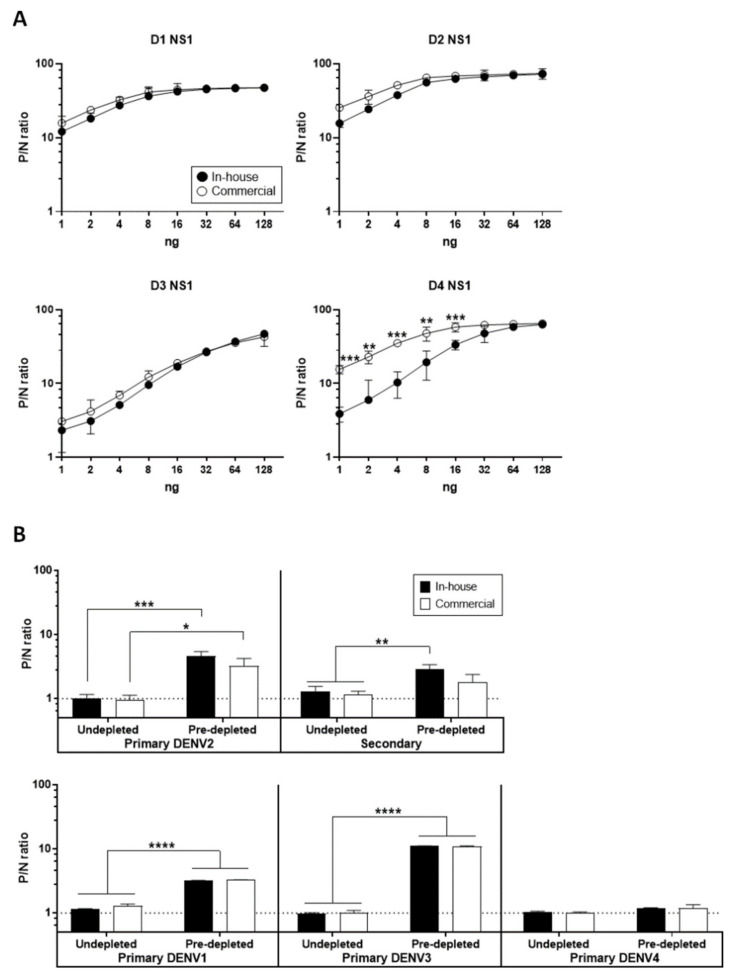
Antigenicity of in-house-produced and commercial DENV NS1 proteins. (**A**) Both in-house-produced and commercial D1 to 4 NS1 antigens were titrated at varying amounts in an Ag–ELISA and detected with serotype-specific murine hyperimmune sera to compare the antigenicity of proteins from different sources. (**B**) NS1 immunoglobulin G-antibody capture enzyme-linked immunosorbent assay (GAC–ELISA) showing similar antigenicity of both in-house-produced and commercial NS1 antigens using four sera with primary DENV1 to 4 infections and one with secondary infection. NS1 GAC–ELISA was performed with or without predepletion of serum anti-premembrane/envelope (prM/E) antibodies using a combination of DENV serotypes 2 + 3 virus-like particle (VLP) antigens. The dotted line indicates the positive-to-negative (P/N) ratio of negative control serum. All data were obtained from three independent experiments with duplicates, and the standard deviations are indicated by error bars. Means were analyzed by one-way analysis of variance (ANOVA) and statistical significance is indicated with asterisk (* *p* < 0.05; ** *p* < 0.01; *** *p* < 0.001; **** *p* < 0.0001).

Furthermore, we used an Ag–ELISA to determine the antigenicity of NS1 proteins. As shown in Figure 2A, using dengue serotype-specific MHIAF as detector antibodies, the DENV1–3 NS1 proteins from both sources also showed similar antigenicity. In contrast, the in-house-produced DENV4 NS1 protein showed significantly lower binding reactivity to the MHIAF at lower concentrations (from 16 ng to 1 ng). This decreased binding could be due to the strain difference giving rise to a heteroclitic reactivity similar to other studies before [38,39], particularly for the commercial DENV4 NS1, which has a higher affinity for the MHIAF than does the immunogen from where it was raised. Note that the anti-DENV4 MHIAF was raised from the homologous DENV4 strain H241 used to produce our in-house DENV4 NS1 protein, whereas the commercial DENV4 NS1 protein was produced using the heterologous DENV4 strain Dominica. Based on the antigen titration curve, P/N ratios generally started to plateau at 16 ng of protein, indicating the optimal concentration for antigen detection. Hence, 16 ng of NS1 protein from each serotype were used in the follow-up experiments. To further confirm the antigenicity of these NS1 proteins, we randomly selected five sera (from Table 1) representing primary and secondary infections and tested them with NS1 GAC–ELISA. As shown in Figure 2B, the NS1 proteins can also be detected by anti-NS1 IgG antibodies present in the sera of individuals with primary DENV1 to 4 infections and secondary infection, indicating that the in-house-produced NS1 proteins have shared antigenicity with the commercial proteins. Consistent with our previous findings [28,29], predepletion of serum anti-prM/E antibodies with VLP antigens enhanced the positive detection of anti-NS1 antibodies in both dengue patients’ sera.

### 3.2. Optimal Condition for NS1 GAC–ELISA

**Figure 3:**
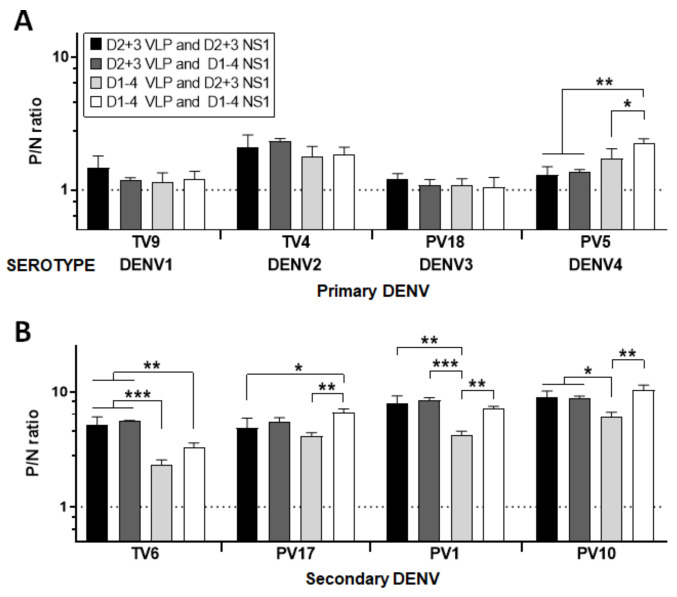
Determination of the optimal antigen combinations for NS1 GAC–ELISA. Anti-prM/E antibodies in community serum samples were predepleted using a combination of DENV2 + 3 VLP antigens only or DENV1–4 VLP antigens before performing the GAC–ELISAs. Both combination strategies were similarly applied for the NS1 antigens in the subsequent GAC–ELISAs of sera coming from four volunteers with primary (**A**) DENV1 to 4 infections and from four volunteers with secondary (**B**) DENV infections. The dotted lines indicate the P/N ratio of negative control serum. All data were obtained from three independent experiments with duplicates, and the standard deviations are indicated by error bars. Means were analyzed by one-way ANOVA and statistical significance is indicated with asterisk (*, *p* < 0.05; **, *p* < 0.01; ***, *p* < 0.001).

Previously, we have shown that using a double homologous serotype combination of DENV2 + 3 VLP and NS1 proteins in NS1 GAC–ELISA is sufficient to detect dengue serum anti-NS1 IgG antibodies [28]. To evaluate the optimal NS1 GAC–ELISA condition further, we compared double or quadruple homologous and heterologous serotype combinations of VLPs and NS1 antigens for the predepletion of anti-prM/E antibodies and detection of anti-NS1 IgG antibodies, respectively, as follows: (1) DENV2 + 3 VLPs and DENV2 + 3 NS1 proteins; (2) DENV2 + 3 VLPs and DENV1–4 NS1 proteins; (3) DENV1–4 VLPs and DENV2 + 3 NS1 proteins; and (4) DENV1–4 VLPs and DENV1–4 NS1 proteins. To test these antigen combinations, we selected eight sera (with FRμNT90-confirmed serostatus) from the community serum panel, including four sera with primary DENV1 to 4 infections and four sera with secondary infections. As shown in Figure 3A, there were no differences observed in any of the conditions among the sera with primary DENV1, 2, and 3 infections. In primary DENV4 infection serum, condition (4) gave a significantly higher P/N ratio, although the other three conditions sensitively detected anti-NS1 IgGs, as demonstrated by higher P/N ratios than the negative control serum. On the other hand, conditions (1) and (2) generally gave the optimum P/N ratios among sera with secondary infections (Figure 3B). Thus, subsequent NS1 GAC–ELISAs were performed using condition (1), as carried out in our previous studies [28,30].

### 3.3. Overall Performance of Diagnostic Assays

**Figure 4:**
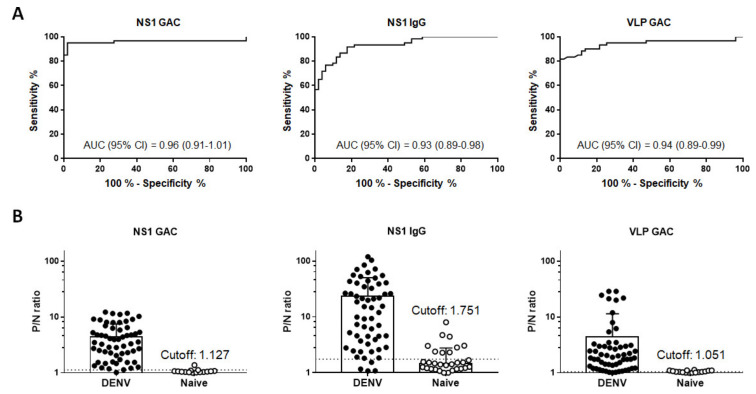
Diagnostic performances of NS1 GAC-, indirect NS1 IgG, and VLP GAC–ELISAs. DENV2 + 3 VLP and NS1 antigens were used in the predepletion and subsequent NS1 GAC–ELISA. Combined DENV1–4 NS1 antigens were used in the indirect NS1 IgG ELISA. DENV2 + 3 VLP antigens were also used in the VLP GAC–ELISA. (**A**) Comparison of the diagnostic performances between NS1 GAC-, indirect NS1 IgG and VLP GAC–ELISAs, as depicted by the fitted receiver operating characteristic (ROC) curves based on P/N ratio values from 60 late convalescent-phase DENV-infected sera and 51 negative control sera. (**B**) The optimal P/N ratio cutoffs (dotted lines) were determined by the magnitude of the positive likelihood ratio (LR^+^) using the formula: LR^+^ = sensitivity/(100 − specificity). All data were obtained from two independent experiments with duplicates. Error bars indicate standard deviations of means.

To compare the diagnostic accuracies of NS1 GAC- and indirect NS1 IgG ELISAs, we simultaneously tested these two assays against a total of 111 community serum specimens consisting of 60 confirmed DENV infections and 51 non-dengue negative controls. Comparative ROC curve analysis revealed comparable high accuracies between NS1 GAC–ELISA (AUC 0.96, 95% CI 0.91–1.01) and NS1 IgG indirect ELISA (AUC 0.93, 95% CI 0.89–0.98) without statistical significance (Figure 4A and Table 2). These results were further compared against the performance of VLP GAC–ELISA. As shown in Figure 4A and Table 2, the VLP GAC–ELISA also demonstrated a comparable accuracy (AUC 0.94, 95% CI 0.89–0.99) against the two NS1 ELISAs. Finally, using the information generated from the ROC curve analysis, the LR^+^ ratio of each assay was calculated to determine the optimal P/N ratio cutoff for positive detection. The optimal P/N ratio cutoffs were set at 1.127, 1.751, and 1.051 for NS1 GAC–ELISA, indirect NS1 IgG ELISA, and VLP GAC–ELISA, respectively (Figure 4B). Comparison between NS1 MAC–, indirect NS1 IgM, and VLP MAC–ELISAs were also performed using 31 patients’ sera and the 51 non-dengue negative control sera. Comparative ROC curve analysis revealed that the NS1 and VLP MAC–ELISAs had significantly higher diagnostic accuracies than the indirect NS1 IgM ELISA (*p* < 0.0001, Figure S1A and Supplementary Table S2).

### 3.4. Limits of Anti-NS1 IgG Detection

**Figure 5:**
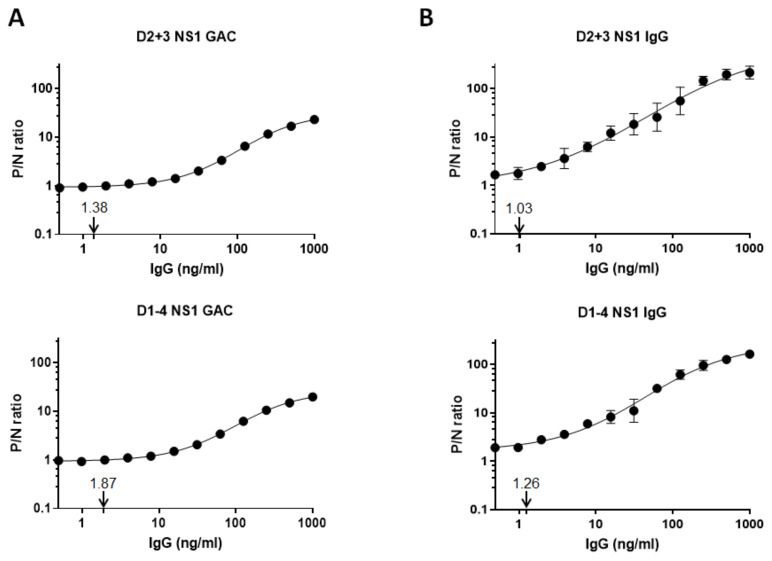
Limits of detection of NS1 GAC- and indirect NS1 IgG ELISAs. Purified pan-flavivirus anti-NS1 chimeric mhFL0221 monoclonal IgG and negative control IgG were twofold titrated at varying concentrations to compare the limits of detection of (**A**) NS1 GAC- (**B**) and indirect NS1 IgG ELISAs using a combination of DENV2 + 3 or DENV1–4 NS1 antigens. The P/N ratio is plotted against anti-NS1 IgG concentration (ng/mL). The limit of detection, indicated with an arrow, is the estimated minimum detectable IgG concentration, as determined by a four-parameter logistic log fit model of the standard curve data. Error bars indicate standard deviations of means.

To compare the sensitivities of NS1 GAC- and indirect NS1 IgG ELISAs, we purified a pan-flavivirus anti-NS1 MAb (chimeric mhFL0221) and polyclonal negative control serum IgGs. Using a combination of DENV2 + 3 or DENV1–4 NS1 antigens, both monoclonal and polyclonal negative control IgGs were titrated twofold to generate a 12-point assay standard curve ranging from 0.5 to 1000 ng/mL. Based on the 4-PL regression analyses, DENV2 + 3 and DENV1–4 NS1 GAC–ELISAs had estimated MDCs of 1.38 and 1.87 ng/mL, respectively (Figure 5A). The indirect DENV2 + 3 and DENV1–4 NS1 IgG ELISAs also had comparable MDCs of 1.03 and 1.26 ng/mL, respectively (Figure 5B). These results demonstrated that both assays are highly sensitive in detecting anti-NS1 IgG regardless of the NS1 antigen combinations and further support that DENV2 + 3 NS1 antigens are sufficient for IgG detection.

### 3.5. Determination of Serostatus Using a Composite Reference Standard

The gold standard used to confirm the serostatus of presumed dengue infections is the NT. However, this test is very time consuming and labor intensive and requires skilled personnel and a biocontainment facility to handle live viruses. Utilizing an ELISA, which yields a good correlation with NT [23,40,41], to detect anti-E or -NS1 antibodies is a better alternative; however, choosing the optimal cutoff involves careful balancing between sensitivity and specificity that does not always give 100% accuracy. Therefore, to develop a reliable diagnostic approach without the need for NT, we combined the test results of indirect NS1 IgG, NS1 GAC-, and VLP GAC–ELISAs for each specimen to create a composite reference standard. Each assay’s sensitivity and specificity, including the composite reference standard, defined as the detection of IgG antibodies in two or more tests, were evaluated against NT (Table 2). NS1 GAC–ELISA demonstrated the highest sensitivity and specificity among the three ELISAs at 95% (95% CI, 86.08–98.96) and 98.04% (95% CI, 89.55–99.95), respectively. Comparable sensitivities were also observed between indirect NS1 IgG and VLP GAC–ELISAs at 91.67% (95% CI 81.61–97.24) and 90% (95% CI 79.49–96.24), respectively; nonetheless, the specificities were relatively lower at 82.35% (95% CI 69.13–91.60) and 86.27% (95% CI 73.74–94.30), respectively. When positive results in two ELISA tests were used as a composite reference standard, the sensitivity and specificity were similar to NS1 GAC–ELISA. Moreover, a 100% (95% CI 96.38–1.00) specificity was achieved when positive results in all three ELISA tests were used as the standard for serostatus determination. Overall, results indicate that a serum specimen can be classified as true dengue positive if it is positive in at least two ELISA tests.

## 4. Discussion

To date, no commercial kit is available to detect anti-NS1 antibodies among patients with dengue or other flaviviral infections. Most commercially available test kits are based on detecting specific antibodies against the immunodominant structural E protein. However, cross reactivity of antibodies frequently confounds the interpretation of results in the diagnosis of flaviviral infections, especially in areas where two or more flaviviruses cocirculate. Patients with previous flavivirus infections (e.g., TBEV, WNV, or YFV infections) and vaccinated individuals (e.g., DENV, JEV, YFV) may develop cross-reactive antibodies later when a secondary immune response is acquired from sequential flavivirus infections or vaccination. An NS1-based diagnostic tool can facilitate precise identification of flavivirus infections with less cross reactivity among different serocomplexes. It can be used to differentiate the vaccine-induced flavivirus-specific antibodies, as previously shown in TBEV [16], JEV [17,18,19], and DENV [21,22] infections.

While a comprehensive review on the performances of various commercially available E-based ELISA kits for both IgM and IgG detection (E-MAC/GAC–ELISA) [42,43] can be found in the literature, relatively fewer studies, summarized in Table 3, have focused on detecting anti-NS1 antibodies for determining dengue serostatus despite its potential utility as diagnostic biomarkers of natural infection. The lack of interest in anti-NS1 antibody detection as a diagnostic tool in flavivirus serodiagnosis may in part be due to the limited information on the kinetics and persistence of anti-NS1 antibodies in infected humans. However, previous studies have reported that high NS1 IgGs could still be detected beyond three years postsymptom onset [22,44]. Consequently, no direct comparison of the accuracy and sensitivity has been made for NS1 GAC- and indirect NS1 IgG ELISAs.

To our knowledge, the study described here is the first to demonstrate that both NS1 GAC- and indirect NS1 IgG ELISAs have comparable diagnostic accuracies and sensitivities (Figure 4A and Table 2) in detecting anti-NS1 IgGs from a community serum collection. Using the purified pan-flavivirus anti-NS1 MAb mhFL0221 instead of a purified polyclonal anti-NS1 IgGs from pooled sera of dengue antibody-positive individuals, we also have demonstrated that both NS1-based assays have comparable limits of IgG detection to as low as ~1–2 ng/mL (Figure 5), which could be closely similar to the threshold (2.33 EU/mL) reported by Nascimento et al. [21]. While MAb mhFL0221 recognizes only a single epitope on the NS1 protein, the calculated MDC could estimate the lowest detection threshold for the polyclonal serum IgGs recognizing multiple NS1 epitopes.

This study also confirms that indirect anti-NS1 ELISA has poor diagnostic performance (AUC 0.56, 95% CI 0.43–0.69) in detecting IgM antibodies in acute-phase to early convalescent-phase DENV infected sera (Figure S1A). This finding could be due to the relatively high abundance of anti-prM/E antibodies in DENV infected individuals, as reported in previous studies [14,47,48]. Additionally, the reduced sensitivity of indirect NS1 IgM ELISA could be due to the competition between two antibody isotypes, i.e., IgM and IgG, for the same antigenic sites.

Dengvaxia^®^, the only commercially available dengue vaccine, is a live-attenuated chimeric yellow fever–DENV tetravalent dengue vaccine (CYD–TDV) [49] known to have a low efficacy among dengue naïve children or children younger than six years old [50,51]. Further studies also revealed an increased risk of severe dengue in seronegative vaccine recipients suggesting that vaccination mimics a primary infection, sensitizing the naïve recipient to antibody-dependent enhancement (ADE) of disease following subsequent natural DENV infection [52,53]. For these reasons, it is only recommended for individuals living in dengue-endemic countries and with laboratory-confirmed DENV exposure [54,55]. Therefore, it becomes crucial to serologically determine the immune status before receiving Dengvaxia^®^ vaccination because a naïve individual has a higher risk of developing a severe disease during subsequent exposure to wild-type DENV. Since each antibody assay has its respective sensitivity and specificity, no assay can determine the immune status with 100% accuracy. A dengue test has been recommended to have a sensitivity and specificity of at least 90%, with the desired sensitivity of 95% and specificity of 98% [56]. In the context of prevaccination screening, a rapid diagnostic test (RDT, i.e., lateral flow immunochromatographic assay) is suitable for providing results at the point of care. Nonetheless, currently available dengue RDTs have low to moderate sensitivities (40–75%) despite their high specificity (>95%) [43]. In this study, it is worth noting that our NS1 GAC–ELISA meets both the minimum and desired accuracies and has comparable accuracy as the indirect NS1 IgG ELISAs used by Nascimento et al. [21] and Tyson et al. [24]. Likewise, our result is comparable to the sensitivity and specificity (95.2% and 93.4%, respectively) of the E-based indirect ELISA kit (Panbio^®^ Dengue IgG Indirect ELISA) used by Lopez et al. [57] in determining dengue serostatus in a cohort of school children, aged 9–14 years old, in the Philippines.

Here, we also developed a composite reference standard (Table 2) to increase further our assay’s specificity, which is very important in avoiding erroneous vaccination. Our current results showed that a serum specimen could be classified as true dengue positive if it yields positive results in at least two ELISA tests, with a high sensitivity of 95%. We further found that a 100% specificity can be achieved when all three ELISAs are serially tested, and the negative results are obtained from all three assays. Arguably, implementing the composite reference assays as screening tools could increase the logistics and cost during assessments of vaccine eligibility, adding further constraints, particularly in developing countries where some patients can hardly afford a dengue test. However, this is the best option available at this time before a new innovative assay (e.g., biosensor-based assay) is developed that can replace ELISAs or RDTs as point-of-care tests while also meeting the World Health Organization’s REASSURED (real-time connectivity, ease of specimen collection, affordable, sensitive, specific, user-friendly, rapid, equipment-free, delivered) criteria [58,59].

This study has a few limitations. First, analyses were only performed using a small sample size of archived specimens from individuals with DENV natural infections. We could not test and compare the diagnostic utility of the NS1-based ELISA methods in differentiating vaccine-induced immunity from natural infection due to difficulty acquiring sera from vaccinated individuals. Second, we could not test and compare the ability of the NS1-based ELISA methods to discriminate DENV infection from other flaviviral infections due to difficulty acquiring serum samples from said infections. Nonetheless, our previous studies have demonstrated that the DENV2 + 3 NS1 MAC/GAC–ELISA had lower cross reactivities, compared to VLP MAC/GAC–ELISA when tested against a limited number of sera with WNV [28] and ZIKV [30] infections, as supported by other studies using DENV1–4 indirect NS1 IgG ELISA with WNV [21] and primary ZIKV [24] infections. To obtain an adequately larger sample size of sera for rigorous validation of our results, including post-dengue vaccination and natural infection (with sufficient representation of the different serotypes) and sera from other flaviviral infections such as JEV and ZIKV, we are currently collaborating with partner laboratories in Southeast Asia where cotransmission between DENV, JEV, and ZIKV is endemic. 

In summary, with different ELISA formats in detecting NS1 IgG, our study suggests that both NS1 GAC- and indirect NS1 IgG ELISAs have comparable limits for detecting anti-NS1 antibodies and diagnostic accuracies. Finally, using a composite reference standard could establish an individual’s serostatus with increased sensitivity and specificity of 95% and 100%, respectively.

## Figures and Tables

**Table 1:** Characteristics of the serum panels used in this study.

Type of Assay	Serum Panel	Classification	No. of Samples	Confirmatory Test
NS1 ^a^ and VLP ^b^ GAC ^c^- and Indirect NS1 IgG ELISA	Community sera			
	Primary DENV1	Late convalescent ^f^	7	FRµNT90 *
	Primary DENV2	Late convalescent	10	FRµNT90
	Primary DENV3	Late convalescent	2	FRµNT90
	Primary DENV4	Late convalescent	1	FRµNT90
	Secondary infections	Late convalescent	41	FRµNT90
NS1 and VLP MAC ^d^- and Indirect NS1 IgM ELISA ^e^	Patient sera			
	Primary DENV1	Acute ^g^	5	RT–PCR ^#^
		Early convalescent ^h^	7	
		Late convalescent	5	
	Primary DENV2	Early convalescent	2	RT–PCR
	Primary DENV3	Acute	1	RT–PCR
		Early convalescent	11	
NS1 and VLP GAC/MAC- and Indirect NS1 IgG/IgM ELISA	Naïve sera	n/a	51	FRµNT90

* 90% focus reduction microneutralization test (FRµNT90). ^#^ Reverse-transcription polymerase chain reaction. ^a^ Nonstructural protein 1 (NS1). ^b^ Virus-like particle (VLP). ^c^ Immunoglobulin G-antibody capture (GAC). ^d^ Immunoglobulin M-antibody capture (MAC). ^e^ Enzyme-linked immunosorbent assay (ELISA). ^f^ >3 months to years postsymptom onset (PSO). ^g^ <7 days PSO. ^h^ <3 months PSO. n/a, not applicable.

**Table 2:** Comparison of NS1 GAC-, indirect NS1 IgG, VLP GAC–ELISAs, and composite reference standards with FRµNT in the determination of dengue serostatus.

		FRµNT				
Test	Result	Positive	Negative	AUC ^#^ (95% CI)	% Sensitivity (95% CI)	% Specificity (95% CI)
NS1 GAC	Positive	57	1	0.96 (0.91–1.01) ^a,b^	95 (86.08–98.96)	98.04 (89.55–99.95)
	Negative	3	50			
NS1 IgG	Positive	55	9	0.93 (0.89–0.98) ^c^	91.67 (81.61–97.24)	82.35 (69.13–91.60)
	Negative	5	42			
VLP GAC	Positive	54	7	0.94 (0.89–0.99)	90 (79.49–96.24)	86.27 (73.74–94.30)
	Negative	6	44			
Composite reference standard 1 *	Positive	57	2	n/a	95 (86.08–98.96)	96.08 (86.54–99.52)
	Negative	3	49			
Composite reference standard 2 **	Positive	51	0	n/a	85 (73.43–92.90)	100 (96.38–1.00)
	Negative	9	51			

* Positive in at least two ELISA tests. ** Positive in all three ELISA tests. ^#^ Area under the curve (AUC). ^a^ NS1–GAC versus NS1 IgG, *p* = 0.2416. ^b^ NS1–GAC versus VLP–GAC, *p* = 0.3855. ^c^ NS1 IgG versus VLP–GAC, *p* = 0.7822. n/a, not applicable.

**Table 3:** Studies detecting anti-DENV NS1 IgG using various serological assays.

Reference	Type of Assay	Serum Dilution	Antigens Used	Gold Standard	% Sensitivity (95% CI) (or % Positive Rate)	% Specificity (95% CI)	MDC ^a^
[21]	Indirect IgG ELISA	1:50	DENV1–4 NS1	PRNT ^c^	91.89 (83.11–96.54)	84.62 (65.85–94.47)	2.33 EU/ml
[24]	Indirect IgG ELISA	1:400	DENV1–4 NS1	RT–PCR ^d^	95.6 (91.40–97.80)	89.5 (84.10–92.30)	n/a
[45]	Indirect IgG ELISA	1:400	DENV1 NS1	RT–PCR	81.30–95.80 positive rate	n/a ^e^	n/a
[46]	MIA ^b^	1:100	DENV1–4 NS1	PRNT	89 (54.33–99.99)	86 (46.65–99.47)	n/a

^a^ Minimum detectable concentration. ^b^ Microsphere immunofluorescence assay. ^c^ Plaque reduction neutralization test. ^d^ Reverse transcription–polymerase chain reaction. ^e^ n/a, not available.

## Data Availability

The data in this research may be made available upon request to the corresponding author.

## Supplementary Materials

The following are available online at https://www.mdpi.com/article/10.3390/diagnostics11050741/s1, Figure S1: Diagnostic performances of NS1 MAC-, indirect NS1 IgM, and VLP MAC-ELISAs, Table S1: Oligonucleotides used in site-directed mutageneses (SDM) and plasmid constructions, Table S2: Comparison of NS1 MAC-, indirect NS1 IgM, and VLP MAC-ELISAs with RT-PCR in the determination of dengue serostatus.
