# Supplementary material for: Comparable Accuracies of Nonstructural Protein 1- and Envelope Protein-Based Enzyme-Linked Immunosorbent Assays in Detecting Anti-Dengue Immunoglobulin G Antibodies
Source: Diagnostics (Basel). 2021 Apr 21;11(5):741. doi: 10.3390/diagnostics11050741 (PMC8143319; doi:10.3390/diagnostics11050741)
Supplement: Supplementary file 1 [file diagnostics-11-00741-s001.zip › diagnostics-1133528-supplementary.pdf]

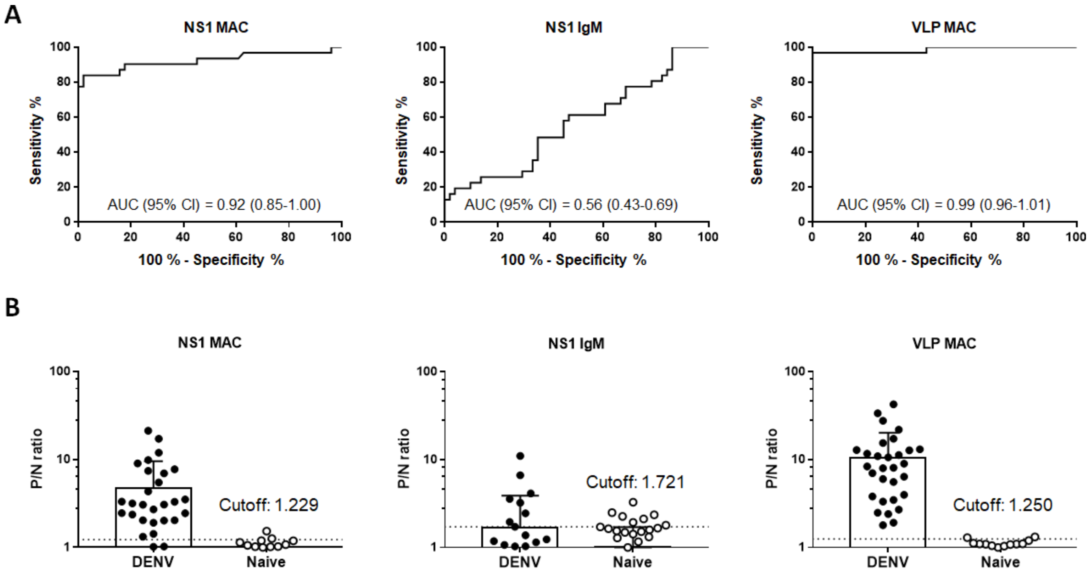

Figure S1. Diagnostic performances of NS1 MAC-, indirect NS1 IgM, and VLP MAC-ELISAs. DENV2+3 VLP and NS1 antigens were used in the pre-depletion and subsequent NS1 MAC-ELISA. Combined DENV1-4 NS1 antigens were used in the indirect NS1 IgM ELISA. DENV2+3 VLP antigens were also used in the VLP MAC-ELISA. (A) Comparison of the diagnostic performances between NS1 MAC-, indirect NS1 IgM, and VLP MAC-ELISAs as depicted by the fitted ROC curves based on P/N ratio values from 31 primary acute-phase to early convalescent-phase DENV-infected sera and 51 negative control sera. (B) The optimal P/N ratio cutoff (dotted lines) were determined by the magnitude of LR^+ using the formula: LR^+ = sensitivity/(100 – specificity). All data were obtained from two independent experiments with duplicates. Error bars indicate standard deviations of means.

Table S1. Oligonucleotides used in site-directed mutageneses (SDM) and plasmid constructions.

Primer	Primer sequence (5′-3′)[a]	Used for
D1 NS1-His	GGTCTCTGCTCACCACCACCACCACCACTGATGAGCGGCCGCTC	SDM, insertion of 6xHis tag at D1 NS1 C-terminal
D2 NS1-His	GGTCACAGCCCACCACCACCACCACCACTGATGAGCGGCCGCTC	SDM, insertion of 6xHis tag at D2 NS1 C-terminal
D3 NS1-His	AGCCTCAGCACACCACCACCACCACCACTGATGAGCGGCCGCTC	SDM, insertion of 6xHis tag at D3 NS1 C-terminal
D4 NS1-His	GGTATCGGCCCACCACCACCACCACCACTGATGAGCGGCCGCTC	SDM, insertion of 6xHis tag at D4 NS1 C-terminal
SH200-NS1-His F	AGTCATCGCTATTACCATGGTAAATGGCCCGCCTGGCT	SH200 donor plasmid cloning, amplification of NS1-His insert
SH200-D1 NS1-His R	CTGGCAACTAGAAGGCACAGGCGAGGCTGATCAGCG	SH200 donor plasmid cloning, amplification of NS1-His insert
SH200-F	CCTGTGCCTTCTAGTTGCCAGCCATCTGTTGTTTGC	SH200 donor cloning plasmid, amplification of vector backbone
SH200-R	CCATGGTAATAGCGATGACTAATACGTAGATGTACTGCCAAGTAGGAAAG	SH200 donor plasmid cloning, amplification of vector backbone

[a]6xHis tag sequences are underlined.

Table S2. Comparison of NS1 MAC-, indirect NS1 IgM, and VLP MAC-ELISAs with RT-PCR in the determination of dengue serostatus.

Test	Result	RT-PCR		AUC (95% CI)	% Sensitivity (95% CI)	% Specificity (95% CI)
		Positive	Negative			
NS1 MAC	Positive	26	2	0.92 (0.85 - 1.00)	83.87 (66.27-94.55)	96.08 (86.54-99.52)
	Negative	5	49			
NS1 IgM	Positive	8	7	0.56 (0.43 - 0.69)*	25.81 (11.86-44.61)	86.27 (73.74-94.30)
	Negative	23	44			
VLP MAC	Positive	30	2	0.99 (0.96 - 1.01)	96.77 (83.30-99.92)	96.08 (86.54-99.52)
	Negative	1	49			

*$p < 0.0001$, significantly different compared to NS1 and VLP MAC.
